# Nutritional and lifestyle supportive care recommendations for management of obesity with GLP-1 - based therapies: An expert consensus statement using a modified Delphi approach

**DOI:** 10.1016/j.obpill.2025.100228

**Published:** 2025-11-11

**Authors:** J.L. Sievenpiper, J. Ard, M. Blüher, W. Chen, J.B. Dixon, A. Fitch, L. Gigliotti, K. Khunti, A. Lecube, M.E.J. Lean, B. Mittendorfer, A.F.H. Pfeiffer, D.H. Ryan, T. Vilsbøll, L.F. Van Gaal

**Affiliations:** a Departments of Nutritional Sciences and Medicine, Temerty Faculty of Medicine, University of Toronto, Toronto, ON, Canada; b Department of Epidemiology and Prevention, Wake Forest School of Medicine, Winston-Salem, NC, USA; c Helmholtz Institute for Metabolic, Obesity and Vascular Research (HI-MAG) of the Helmholtz Zentrum München at the University of Leipzig and University Hospital Leipzig, Leipzig, Germany; d Department of Clinical Nutrition, Peking Union Medical College Hospital, Chinese Academy of Medical Sciences and Peking Union Medical College, No. 1 Shuai fu yuan Wang fu jing, Dong Cheng District, Beijing, 100730, China; e Iverson Health Innovation Research Institute, Swinburne University of Technology, Melbourne, Australia; f knownwell Health, Boston, MA, USA; g Irvine, CA, USA; h Diabetes Research Centre, University of Leicester, Leicester, UK; i Endocrinology and Nutrition Department, Vall d'Hebron University Hospital, VHIR, Barcelona, Spain; j Centro de Investigación Biomédica en Red de Diabetes y Enfermedades Metabólicas Asociadas (CIBERDEM), Madrid, Spain; k Human Nutrition, School of Medicine, Dentistry and Nursing, College of Medical, Veterinary and Life Sciences, University of Glasgow, Glasgow, UK; l Departments of Medicine and Nutrition & Exercise Physiology, University of Missouri School of Medicine, Columbia, MO, USA; m Charité - Universitätsmedizin Berlin, Klinik für Endokrinologie, Stoffwechsel- und Ernährungsmedizin, Berlin, Germany; n Pennington Biomedical Research Center, Louisiana State University, Baton Rouge, LA, USA; o Clinical Research, Steno Diabetes Center Copenhagen, University of Copenhagen, Herlev, Denmark; p Department of Clinical Medicine, Faculty of Health and Medical Sciences, University of Copenhagen, Copenhagen, Denmark; q Department of Endocrinology, Diabetology and Metabolism, Antwerp University Hospital, University of Antwerp, Antwerp, Belgium; r Division of Endocrinology and Metabolism, Department of Medicine, St. Michael's Hospital, Toronto, ON, Canada; s Li Ka Shing Knowledge Institute, St. Michael's Hospital, Toronto, ON, Canada

**Keywords:** Liraglutide, Nutrition, Physical activity, Semaglutide, Tirzepatide, Weight management

## Abstract

**Background:**

Liraglutide, semaglutide and tirzepatide have transformed the management of obesity. However, dose-related gastrointestinal effects, obesity-associated nutritional insufficiencies, and poor long-term adherence may limit their long-term health benefits. Despite a recent joint advisory summarizing nutritional and lifestyle supportive care priorities with these therapies, there is still a significant lack of direct evidence to guide clinical practice, making consensus-based recommendations necessary.

**Methods:**

The consensus statement development was based on an initial scoping review that included searching PubMed, Embase, Web of Science, Cochrane, and Medline for relevant scientific publications from January 1, 2021 through June 30, 2025. An international multidisciplinary panel consisting of physicians, clinical researchers, and dietitians employed a modified Delphi process to develop clinical practice recommendations for nutritional and lifestyle strategies that may assist people on glucagon-like peptide 1 based therapies (GBT) in optimizing treatment experience and improving health outcomes.

**Results:**

A total of 52 consensus statements were developed, outlining key considerations for the practical management of obesity and associated complications with GBTs, with a focus on nutritional factors in relation to obesity, body composition, physical activity, and the management of common gastrointestinal symptoms such as nausea, vomiting, diarrhoea, and constipation. The consensus statements include practical strategies supporting the weight loss journey, from before starting a GBT, during the weight loss and weight maintenance phases, and in case of GBT discontinuation. The statements were primarily derived from indirect evidence, including from existing evidence and established guidelines for nutrition therapy in bariatric medicine and relevant clinical experience.

**Conclusions:**

These expert consensus recommendations offer healthcare professionals practical guidance on nutritional and lifestyle interventions for patients undergoing GBT-related weight management, complementing current recommendations. Further direct evidence is urgently required to inform and enhance optimal clinical care.

## Graphical abstract

**Figure:**
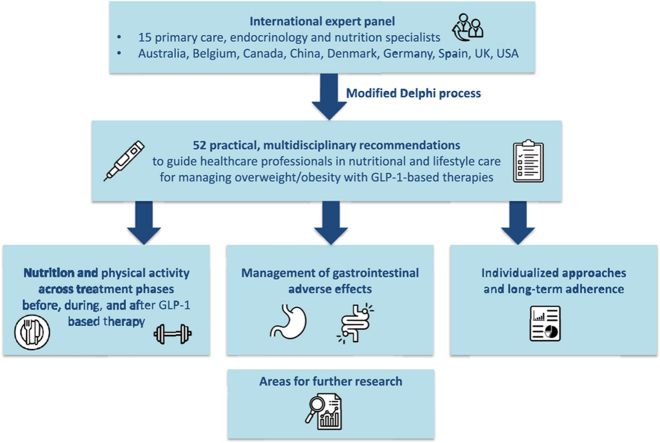

## 1 Introduction

Obesity is a heterogeneous and relapsing progressive chronic disease with high clinical and societal burdens and significant unmet medical needs [[1], [2], [3]]. Obesity treatment guidelines emphasize a stepwise approach, focusing initially on evidence-based nutrition and lifestyle modifications centred on hypocaloric dietary patterns and physical activity regimens, tailored to individual needs, followed by escalation to pharmacotherapy and bariatric surgery [[4], [5], [6], [7]].

The US Food and Drug Administration (FDA) approval for weight management of the first-generation glucagon-like peptide 1 (GLP-1) receptor agonists (RA) liraglutide in 2014 followed by two more potent GLP-1 based therapies (GBT), semaglutide (GLP-1RA) in 2021 and tirzepatide (dual GLP-1/gastric inhibitory polypeptide RA) in 2023 [8,9] have led to a paradigm shift in the management of health conditions related to obesity. Beyond weight management, their indications are broad, including type 2 diabetes, cardiovascular diseases, chronic kidney disease, and obstructive sleep apnea [[10], [11], [12], [13]].

GBTs lead to substantially reduced caloric intake by increasing satiety, suppressing appetite, decreasing hunger, reducing gastrointestinal (GI) and biliary motility, and slowing gastric emptying [14,15]. In clinical trials, semaglutide and tirzepatide have demonstrated mean weight reductions of 15–25 % at 12–36 months, close to that achieved with some forms of bariatric surgery [[16], [17], [18], [19]]. However, lower rates of weight reduction (∼5 %) have been reported in real-world studies of patients with obesity with or without comorbid type 2 diabetes [20,21]. The most common adverse effects (AEs) associated with GBTs are GI, mainly nausea, vomiting, diarrhoea and constipation [22,23]. Most of these AEs occur during the dose escalation phase, are mild to moderate and generally transient [[24], [25], [26], [27], [28], [29], [30], [31], [32], [33], [34], [35]].

Recent calls have highlighted the need for evidence on the long-term impacts of rapid and substantial weight loss, including with GBTs, on muscle health, body composition, and a potential risk of sarcopenia with aging or frailty [[36], [37], [38]]. Some individuals with obesity are at risk of micronutrient deficiencies due to their habitual consumption of an energy-rich, nutrient-poor diet; thus, GBT-mediated potent appetite reduction potentially aggravated by GI adverse events may exacerbate pre-existing micronutrient deficiencies [39].

People with obesity face multiple challenges to effective weight management, including discrimination because of poor understanding of the disease process. Several studies have reported that many people prescribed GBT for obesity do not receive structured dietary and physical activity support due to lack of time and training of primary healthcare professionals, or lack of access to a registered dietitian [[40], [41], [42], [43]]. Large real-world cohorts in the United States of America (USA) and Denmark have reported GBT discontinuation rates of 50 % or more at one year after treatment initiation [20,21,44,45]. As most individuals with overweight/obesity are managed in primary care setting, this paper aims to provide practical recommendations for (but not limited to) non-obesity-specialist healthcare professionals on nutritional and lifestyle strategies in association with GBTs for weight management.

## 2 Methodology

A modified Delphi study [46,47] was conducted with an international expert panel between May and September 2024. The project was overseen by a steering committee (JV and LvG) appointed by the sponsor due to their recognized expertise in nutrition and obesity management, as well as their leadership interest in the study.

A single face-to-face meeting was organised with logistical support from the sponsor; however all consensus content was developed independently by the expert panel, which included endocrinology and nutrition specialists from Australia, Belgium, Canada, China, Denmark, Germany, Spain, the United Kingdom, and the United States. The Chairmen (LvG and JLS), selected by the sponsor for their complementary backgrounds in endocrinology-metabolism, nutrition sciences, obesity care, history of clinical investigation with GBTs and experience with developing guidelines, represented North America and Europe, regions from which most of the panellists would be recruited. The Chairmen independently identified 18 potential panellists based on similar criteria (i.e., expertise in nutrition and obesity, research and clinical care) to establish a pluri-disciplinary group that also included a registered dietitian and an expert in nutrition and exercise physiology. The final expert panel consisted of those 15 members who accepted the invitation to join this project.

### 2.1 Literature review

A scoping review was performed by an independent research company (Sector & Segment, London, United Kingdom [S&S]) appointed by the sponsor, to identify relevant scientific publications from January 1, 2021 (the year of FDA approval of semaglutide for weight management), to June 30, 2025. The databases searched included PubMed, Embase, Web of Science, Cochrane, and Medline, using specific search terms detailed in Supplement Table 1. The review prioritized direct evidence from authoritative clinical practice guidelines adhering to established evidence-based principles, followed by systematic reviews, meta-analyses, randomized controlled trials (RCTs), non-randomized controlled trials (NRCTs), and prospective cohort studies. Panellists considered post-2021 evidence on currently approved weight reduction agents, including semaglutide and tirzepatide, to complement the guideline-derived framework. Additionally, indirect evidence from similar interventions, such as non-GLP-1 therapies, intensive lifestyle interventions, or bariatric surgery targeting comparable weight loss in individuals with and without diabetes, was also evaluated, and the panellists enriched the review by providing complementary publications required to address evidence gaps.

### 2.2 Modified Delphi process

The modified Delphi methodology was employed to develop statements addressing the nutritional, physical activity, and clinical management needs of individuals with obesity undergoing treatment with GBT, segmented into pre-treatment, during treatment, and post-treatment phases. The expert panel met face-to-face in May 2024 to discuss the literature review results and elaborated an initial list of recommendations on nutrition and lifestyle management along the patient journey (before, during, after GBT). Based on this input, the Chairmen (JLS and LvG), with the support of S&S, formulated a first set of statements, which were then anonymously assessed by the expert panel using an online voting platform.

During the first voting round, all panellists independently evaluated each statement, using a nine-point scale ranging from 1 (absolutely disagree) to 9 (absolutely agree). Agreement was defined by a rating score ≥ 7. Consensus thresholds ≥ 67 % agreement (scores ≥ 7 by 10/15 experts) were defined a priori during the planning phase and communicated to panellists before the first round of voting. Following the first round, a virtual meeting was convened to present the anonymized collective results, allowing for discussion and suggested modifications to the statements. Revised statements were prepared by the Chairmen based on this group feedback and distributed for a second round of voting, in which the modified statements were re-evaluated using the same nine-point scale and timeframe. A final virtual meeting was held in September 2024 for the expert panel to discuss the anonymized results from the second round. There was no attrition at any stage of the process, and all experts completed the two voting rounds.

Statements achieving a consensus threshold of ≥ 67 % agreement (scores ≥ 7 by 10/15 experts) were accepted. To represent the diversity of expert opinions on recommendations stemming from extensive clinical experience rather than evidence-based, statements with moderate consensus (scores 7–9 by 60 % to < 67 % of experts, with a median score ≥ 7) were also included.

The degree of consensus (percentage of votes 7–9) and median scores for each statement were calculated and reported. This rigorous and iterative approach ensured transparency and inclusivity in the development of the final recommendations. The study sponsor had no voting rights on statements, no veto, and no influence on inclusion/exclusion of evidence or ranking of statements.

### 2.3 Development of recommendations

To provide clear guidance for primary and non-specialist healthcare professionals, the final recommendations were grouped into seven modules: nutrition, physical activity, treatment considerations before, during, and eventually after GLP-1-based therapy, and management of common therapy-related AEs. The discussion section supplements these recommendations with selected articles published prior to 2021, endorsed by the experts, to provide deeper insights.

## 3 Recommendations

**Fig. 1:**
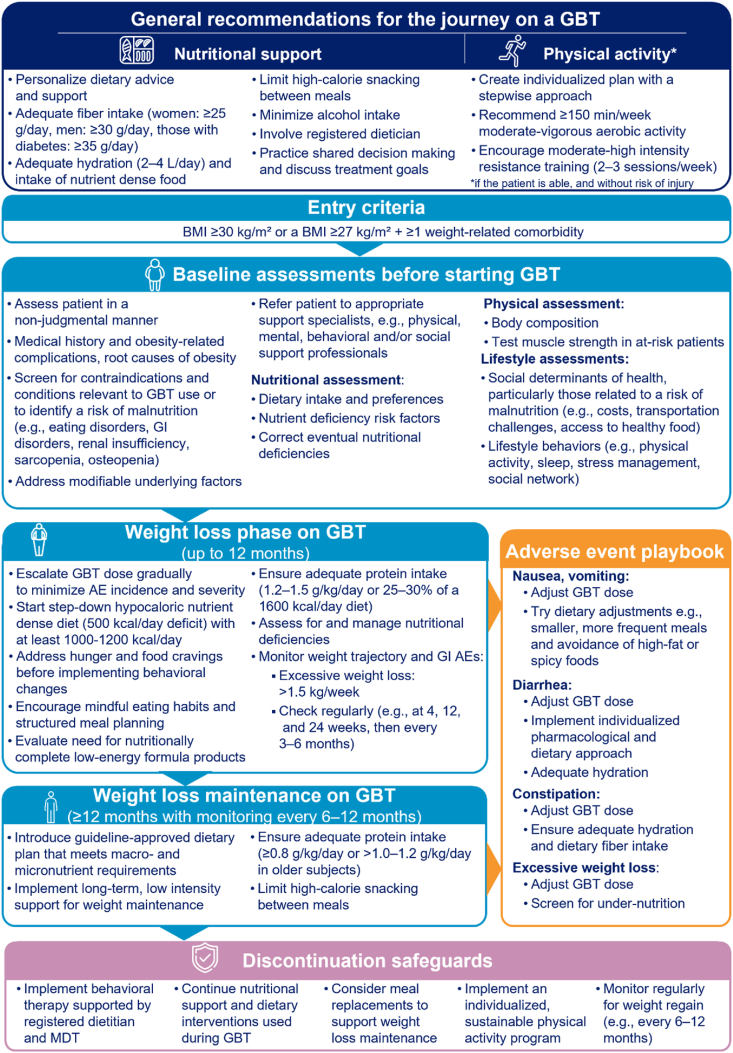
Flowchart outlining key considerations for weight management before, during, and potentially after GBT for weight loss AE, adverse event; BMI, body mass index; GBT, GLP-1 based therapy; GI, gastrointestinal; GLP-1, glucagon-like peptide 1; kcal, kilocalories; L, litre; MDT, multidisciplinary team.

Of the 85 initial statements presented to the experts, consensus was achieved for a total of 52 statements after two rounds of confidential voting. To enhance the applicability of the expert recommendations in clinical practice, recommendations were categorized into seven modules (nutrition, physical activity, treatment considerations before, during, and after GBT, and management of common therapy-related AEs). A list of general nutrition and physical activity statements was considered applicable across all stages of the GLP-1 weight loss journey. While pharmacologic profiles and weight loss outcomes may differ between GBT agents, the nutritional and lifestyle recommendations aim to be applicable across all GBTs approved for weight loss. Most importantly is an individualized approach to each patient's preferences and environment. A flowchart summarizing key considerations for treating individuals with obesity using GBT in primary care is presented in Figure 1, with detailed recommendations outlined in Table 1, Table 2, Table 3, Table 4, Table 5, Table 6, Table 7.

**Table 1:** Nutritional considerations in obesity.

Statements	% scores 7–9	Median score	Evidence level
Nutrition and lifestyle recommendations should be personalized to meet individual values, preferences, and treatment goals to support a dietary approach that is safe, effective, nutritionally adequate, culturally acceptable and affordable for long-term adherence.	100 %	9	Guideline
Nutrition and lifestyle interventions should use a shared decision-making approach to improve overall health, promote a healthy relationship with food, emphasize food quality, consider the social context of eating and promote eating behaviours that are sustainable and realistic for the individual.	100 %	8	Guideline
Most patients should strive to follow a guideline-endorsed dietary pattern.	93 %	8	Guideline
Nutritional management should focus on ensuring adequate nourishment and hydration, preserving lean muscle mass, and minimizing adverse effects in addition to achieving health outcomes for chronic disease risk reduction and quality of life improvements in addition to weight reduction.	93 %	8	Guideline
Alcohol intake should be minimized or discouraged	87 %	8	Expert opinion
In cases of very low energy intake, a high-protein oral nutritional supplement and/or vitamin and mineral supplementation according to established dietary guidelines should be recommended.	87 %	8	Expert opinion
A negative energy balance may have adverse consequences for skeletal health, muscle strength and nutritional health. This highlights the importance of individualizing nutrition interventions that are safe, effective and meet the values and preferences of the patient.	87 %	8	Expert opinion
People living with obesity are at increased risk for micronutrient deficiencies and baseline and follow-up assessments including blood and/or urine biochemistry may help inform recommendations on food intake, vitamin and mineral supplements.	87 %	7	Observational
A registered dietitian should be involved in the assessment, delivery, and evaluation of care wherever possible.	80 %	8	Expert opinion

**Table 2:** Considerations for physical activity and weight loss.

Statements	% scores 7–9	Median score	Evidence level
Exercise prescription must be individualized to the needs, preferences, capacity, corpulence, and health status of each patient to sustain long-term adherence and prevent injuries.	100 %	8	Guideline
An individualized exercise training program based on ≥150 min of moderate to vigorous intensity aerobic activity and resistance training per week supports healthy weight loss.	93 %	7	Guideline
Physical activity should be an integral component of the weight loss plan to decrease cardiovascular risk factors and achieve and maintain an optimal body weight.	87 %	8	Guideline
A high protein intake alone does not increase muscle mass. For preservation of lean body mass (e.g., bone and muscle mass) during weight loss, an exercise training program based on resistance training at moderate-to-high intensity is advised.	80 %	7	Observational
If patients are unable to complete 150 min of supervised exercise per week, a stepwise approach with shared and measurable goals agreed by both the HCP and the patient, such as a structured exercise plan or a minimum of 4000–6000 steps/day is recommended.	73 %	8	Expert opinion
Older, frail, or sedentary patients, or those with sarcopenia may have an increased risk of losing muscle mass during weight loss. Strategies to preserve muscle mass should be considered, including tailored physical activity programs and nutritional interventions.	73 %	7	Expert opinion

HCP, healthcare provider.

**Table 3:** Considerations before starting GBT for weight loss.

Statements	% scores 7–9	Median score	Evidence level
A non-judgmental, stigma-free environment is necessary for an effective assessment of a patient living with obesity.	100 %	9	Guideline
Patients should be prepared for an evolving experience with food throughout the course of their treatment with an individualized approach being applied depending on patient values, preferences, treatment goals and response.	100 %	8	Expert opinion
Identify and address modifiable underlying factors (e.g., mental health/depression, medications such as corticosteroids, anti-depressants, antipsychotics, beta-blockers, insulin, and hormonal abnormalities) that may contribute to weight gain or hinder weight loss.	80 %	8	Guideline
In patients at high risk of losing muscle mass (e.g., patients with sarcopenic obesity), muscle strength should be evaluated using functional tests (e.g., hand grip strength, chair stand test). If available, refer to a specialized centre for body composition assessment.	87 %	7	Expert opinion
Treatment goals should be discussed with the patient along the therapy journey. Beyond the first month it is important to continue monitoring the weight trajectory to identify excessive weight loss (i.e., > 1.5 kg/week)	87 %	7	Expert opinion
A low- to very low-calorie diet (<840 kcal/day) may be prescribed to induce weight loss and lifestyle modifications before initiating a GBT. A step-down approach starting with 1000–1200 kcal/day is commonly advocated.	73 %	7	Expert opinion

GBT, GLP-1 based therapy; kcal, kilocalories.

**Table 4:** Considerations for the weight loss phase.

Statements	% scores 7–9	Median score	Evidence level
Inform the patient to be mindful about the timing and frequency of eating.	93 %	8	Expert opinion
Dietary modifications may be easier to implement after hunger and food cravings are reduced.	87 %	8	Expert opinion
Nutritionally complete low-energy formula products can be used, either temporarily for weight-loss as a ‘total diet replacement’ (i.e., replacing all meals), or ‘partial diet replacement’ (i.e., replacing 1–2 meals/day.	87 %	8	Guideline
Monitoring of adequate hydration, excessive weight loss, and GI adverse effects such as nausea, vomiting or diarrhoea is important during the dose titration phase of GBT.	87 %	8	Expert opinion
During the weight-loss phase on GBT, a protein intake of 1.2–1.5 g/kg actual body weight/day or equivalent to 25–30 % energy on a 1600 kcal/day diet, is recommended.	87 %	7	Expert opinion
Recommended eating behaviours include eating mindfully and ending meals when feeling “comfortably full”.	80 %	7	Expert opinion
A step-down approach starting with a reduced caloric intake by 500 kcal/day below the estimated caloric needs of the individual, with a minimum intake of 1000–1200 kcal/day, is commonly advocated.	80 %	7	Observational
A total dietary fibre intake of ≥ 25 g per day for women and ≥ 30 g per day in men, or ≥ 35 g per day in people with diabetes, is recommended.	73 %	7	Guideline
Adjusting the GBT dose and implementing structured meal plans may be necessary to avoid excessive weight loss.	60 %	7	Expert opinion, limited evidence
For some patients, a structured eating plan could be beneficial with frequent (e.g. 4–6) small meals containing a diet rich in plant-based foods and sources of lean protein.	60 %	7	Expert opinion, limited evidence

GBT, GLP-1 based therapy; kcal, kilocalories.

**Table 5:** Considerations for the weight loss maintenance phase.

Statements	% scores 7–9	Median score	Evidence level
A variety of weight loss approaches can be used equally effectively for weight management (e.g., continuous energy restriction, intermittent fasting) provided that they can be followed and meet nutritional requirements for protein, fat, micronutrient and fibre intake.	93 %	8	Guideline
Following weight loss, long-term support for weight maintenance is recommended.	93 %	8	Guideline
Adequate, high-quality protein consumption is recommended, particularly for patients at risk of obesity-related sarcopenia, to prevent or correct the insufficient protein intake and minimize muscle loss associated with rapid weight loss due to substantially reduced food consumption.	93 %	8	Observational
During weight-loss maintenance on GBT, protein intake should be ≥ 0.8 g/kg actual body weight/day.	93 %	7	Expert opinion
Nutritionally complete low-energy formula products can be used, by replacing 1 meal/day or 3–6 meals/week for longer-term weight-loss maintenance.	87 %	8	Expert opinion
A high intake of dietary fibre from naturally occurring, added or supplemental sources is recommended as part of dietary interventions for weight management. Mixed fibre interventions emphasizing high intakes of both soluble (from fruit, certain vegetables, legumes, oats, barley, psyllium, etc.) and insoluble (from most vegetables, whole wheat, etc.) are recommended, as they have shown cardiometabolic benefits.	80 %	7	Guideline
In between meals, high-calorie nutrient-poor snacking should generally be avoided.	73 %	8	Expert opinion
Water intake between meals and consumption of fibre-rich foods may help to increase the feeling of fullness and reduce over-eating.	73 %	7	Expert opinion
GBT users may be at risk of developing gallstones [91] hence a healthy fat intake of 25–60 g/day for a 1200–1500 kcal/day diet or 35–70 g/day for a 1500–1800 kcal/day diet is recommended to aid the absorption of fat-soluble vitamins and stimulate gallbladder emptying. Foods containing healthy fats include olive oil, nuts, seeds, and fatty fish [[168], [169], [170], [171]].	67 %	7	Expert opinion
People aged 65 years or older with preserved renal function may require a higher protein intake than people younger than 65 years to maintain muscle mass and avoid sarcopenia.	67 %	7	Guideline

GBT, GLP-1 based therapy; kcal, kilocalories.

**Table 6:** Management and mitigation of common side effects associated with GBT for weight loss.

Statements	% scores 7–9	Median score	Evidence level
In cases where the patient experiences nausea or vomiting, HCPs should monitor symptoms, adjust the GBT dose and try individualized dietary approaches to reduce or eliminate these symptoms. Proper hydration should be prioritized.	100 %	8	Guideline
In cases where the patient experiences diarrhoea, an individualized pharmacological and dietary approach along with adequate hydration should be implemented.	100 %	8	Expert opinion
Constipation symptoms occur frequently in people with overweight/obesity and have been reported to last longer than other GBT GI adverse effects. General recommendations include consuming an adequate amount of fibre and increasing the intake of water or other sugar-free liquids. Fiber supplementation (e.g., psyllium) should be considered when sufficient fibre intake cannot be obtained from the diet. Adjustments to fibre intake should be made based on individual patient response and tolerability.	87 %	8	Guideline
Hydration with > 2 L of fluid intake per day is necessary and awareness of adequate hydration is particularly important in cases of exercise, diarrhoea, vomiting and fasting.	87 %	8	Observational
Monitor weight loss, assess loss of appetite, cues to eating, and side effects (e.g., fatigue, constipation). Screen for excessive weight loss which may indicate under-nutrition.	87 %	7	Expert opinion
In most patients who are taking GBT for weight loss, minimizing or preventing nausea is not expected to interfere with the weight loss treatment.	87 %	7	Observational

GBT, GLP-1 based therapy; GI, gastrointestinal; HCP, healthcare provider.

**Table 7:** Considerations if GBT must be discontinued.

Statements	% scores 7–9	Median score	Evidence level
If available, refer to a registered dietitian and implement intensive behavioural therapy with the support of a multidisciplinary team.	100 %	8	Expert opinion
Meal replacements can help as safe and nutrient-relevant solutions. Partial meal replacements (i.e., replacing 1–2 meals/day as part of a calorie-restricted intervention) have been proven to reduce body weight, waist circumference and blood pressure, and improve glycaemic control.	87 %	8	Observational
If a GBT must be interrupted, the dietary intervention should be consistent with the dietary recommendations used when the GBT was first started. Close post-therapy follow-up and repeated lifestyle intervention are recommended to minimize weight regain.	87 %	7	Expert opinion
An individualized, sustainable exercise program consisting of ≥ 150 min per week of moderate to vigorous intensity aerobic activity and resistance training supports weight loss maintenance.	87 %	7	Guideline
Calorie-controlled, protein-rich dietary patterns may offer benefits for preventing weight regain.	67 %	7	Expert opinion

GBT, GLP-1 based therapy.

### 3.1 Nutritional considerations in obesity

The results indicated a high level of expert consensus (80–100 %) for all statements included in the section on nutritional considerations in obesity (Table 1). Nutritional and lifestyle recommendations for individuals with obesity should be highly personalized to align with their values, preferences, and treatment goals [[4], [5], [6],[48], [49], [50], [51]].

In accordance with general dietary guidelines, including American Association of Clinical Endocrinology (AACE) 2016, European Association for the Study of Obesity (EASO) 2022, Canadian Adult Obesity Clinical Practice Guidelines 2022, Diabetes Nutrition Study Group (DNSG)/European Association for the Study of Diabetes (EASD) 2022, a personalized approach to nutrition was strongly endorsed, emphasizing shared decision-making and culturally tailored recommendations to enhance overall health and foster a sustainable and realistic relationship with food [48,[51], [52], [53]]. Several established therapeutic dietary patterns, such as the Mediterranean, Nordic, Vegetarian/Vegan, Low-glycaemic Index, Dietary Approaches to Stop Hypertension, and Portfolio diets, are recommended by clinical practice guidelines and have demonstrated benefits for weight-related outcomes [[4], [5], [6],[54], [55], [56], [57], [58], [59], [60], [61], [62], [63], [64], [65]]. World-wide food-based dietary guidelines reflect the diverse dietary preferences of different countries and have been reviewed elsewhere [[66], [67], [68]], and will therefore not be discussed here.

Minimizing alcohol intake is recommended, as alcohol consumption is inherently likely to impair adherence with diet or medications [69]. This is also in line with a recent systematic review and meta-analysis that found that people who consume large amounts of alcohol had increased odds of overweight and obesity compared to non-alcohol drinkers or light (<14 g/day) alcohol drinkers [70,71].

Effective patient education and communication, guided by the "5 A's" model (Ask, Assess, Advise, Agree, Assist), is essential to encourage behaviour change and support weight loss [72].

Although people with obesity consume an adequate energy intake that supports weight maintenance or positive energy balance [73], they are often paradoxically at an increased risk of malnutrition, particularly micronutrient deficiencies (e.g., vitamin D, vitamin A, thiamine (B1), folate (B9), cobalamin (B12), iron, calcium, and magnesium) [39,48,74]. Causes are multifactorial and may be related to dietary preferences, medications, insufficient access to nutrient-rich foods, changes in the absorption, distribution or excretion of nutrients, and altered micronutrient metabolism due to systemic obesity-mediated inflammation [75,76]. Basic dietary assessment, such as a 24-h or usual dietary recall, and in some cases, biochemical screening, may be considered if there are clinical indications of micronutrient deficiency, but screening is rarely necessary or recommended in routine practice [77]. Dietary advice should strive to ensure nutritional completeness for all vitamins and nutrients. If dietary adherence is doubtful, or weight loss is excessive, micronutrient supplementation is recommended [39,48,73,78]. If available, a registered/certified dietitian or nutritionist should be involved to ensure integrity in the assessment, delivery, and evaluation of care, as this offers a strong, multi-disciplinary, supporting environment with added expertise in individualized nutrition care, to enhance health benefits from weight management and prevent and manage potential nutritional deficiencies [79,80].

Given the substantial interindividual variability in patient responses to GBTs, the expert panel deliberately structured the recommendations to be granular and actionable. Rather than consolidating multiple concepts into fewer, broader statements, the panel opted to offer detailed, actionable recommendations to ensure clarity, enhance practical usability, and allow healthcare providers—particularly those without specialist training—to tailor care more precisely to individual patient needs across various clinical scenarios.

### 3.2 Physical activity and weight loss

A 100 % consensus was reached for physical activity regimens that are individualized to the needs, preferences, capacity, body size, and health status of each patient, designed to sustain long-term adherence and avoid injuries (Table 2).

An individualized physical activity program based on ≥150 min of moderate to vigorous intensity aerobic activity in addition to resistance training per week is recommended to support healthy weight loss and long-term weight and health management in people with obesity. This recommendation is in line with the AACE 2016, EASO 2021 and American College of Sports Medicine 2024 guidelines that recommend that adults with obesity should undertake 150–300 min of moderate-intensity physical activity per week and resistance training 2–3 times per week [53,81,82]. Examples of group and individual exercise regimens that in RCTs have been shown to enhance GBT-mediated weight loss are summarised in Supplement Table 2 [83,84].

RCTs such as SURMOUNT-3 have demonstrated beneficial synergistic anti-inflammatory, cardioprotective and weight loss effects of combining GBT therapy with physical activity [[85], [86], [87]]. Both aerobic activity and resistance training are well-documented for their health benefits, including a modest contribution to weight management, muscle mass growth or preservation, and improved cardiometabolic health [88]. Furthermore, RCT data have shown that combining GBT with exercise for weight loss prevents bone mineral density loss observed with GBT treatment alone [83]. Incorporating a high-protein diet (e.g., 1.2–1.5 g/kg of body weight per day) with an adequate total dietary energy content alongside moderate physical activity may further help to preserve muscle mass and functionality, particularly in older adults and those at risk of obesity-related sarcopenia (i.e. patients with high body mass index (BMI) or large waist circumference, and surrogate parameters for sarcopenia), as outlined in the 2023 European Society for Clinical Nutrition and Metabolism (ESPEN)/EASO sarcopenic obesity diagnostic algorithm [37,[89], [90], [91]].

### 3.3 Before starting a GLP-1 based therapy for weight loss

Weight-based stigma and internalized weight bias significantly harm mental health and may increase risks of depression, anxiety, low self-esteem, social isolation, stress, and substance use. These stigmata also discourage physical activity and promote unhealthy behaviours, thus worsening obesity [92]. The expert panel emphasized creating a stigma-free environment when managing patients with obesity.

Obesity arises from a chronic net energy surplus but has complex, multifactorial causes, including genetics, socioeconomic status, physical inactivity, stress, insufficient hours of sleep, and medications such as antidepressants, antipsychotics, certain antihyperglycemic medications, α2-adrenergic agonists, β2-adrenergic agonists, antiretroviral therapies, anti-epileptics, glucose-lowering drugs and corticosteroids [73,[93], [94], [95]]. When assessing a patient with obesity, identifying and addressing modifiable factors, such as medication use and mental health support, is crucial.

Before initiating GBT, individualized, realistic weight loss goals should be set collaboratively between patients and healthcare professionals. Although GBT clinical trials have reported average weight loss of up to 21 %, with large interindividual variability, real-world outcomes depend on baseline weight and additional support such as diet and exercise [[24], [25], [26], [27], [28], [29], [30], [31],[96], [97], [98]]. According to a prospective study by Wren et al., setting ambitious goals (e.g., > 10 % weight loss) may enhance adherence and outcomes compared to modest goals (mean difference 5.2 kg, 95 % Confidence Interval 5.0–5.4; P < 0.001) [99]. Weight loss targets should also align with health outcomes such as diabetes remission, blood pressure reduction, or cardiovascular risk mitigation [99,100]. It is therefore important to discuss and set realistic and meaningful patient-centric treatment goals for a healthy and attainable weight loss journey. A low- (e.g., 800–1200 kcal/day) to very low- (<800 kcal/day) calorie diet may be prescribed to induce weight loss and lifestyle modifications before initiating a GBT, and a step-down approach starting with 1000–1200 kcal/day is commonly advocated. This approach of sequential treatment strategies appeared to show additive benefits in a trial with liraglutide, in SURMOUNT-3 with tirzepatide, and in STEP-3 with semaglutide [84,87,101]. Recently, a meta-analysis of 33 RCT of more than 12,000 participants also demonstrated benefits of combining lifestyle interventions with GLP-1RAs on weight reduction and cardiometabolic markers [102].

**Fig. 2:**
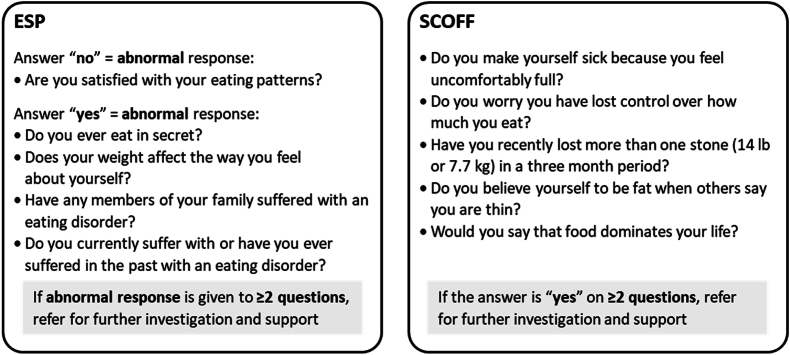
ESP and SCOFF questionnaires for screening for eating disorders The ESP and SCOFF questionnaires are suitable for screening for, but not diagnosing of, eating disorders [105,106] ESP, Eating Disorder Screen for Primary Care; SCOFF, Sick, Control, One stone, Fat, Food.

Although disordered eating appears to be more common in people with obesity than in people with normal weight, it is less frequently diagnosed in people with obesity [103]. GBTs have the potential to worsen eating disorders, so it is therefore important to consider screening for disordered eating or eating disorders prior to prescribing GBT [104]. Eating disorder screening tools suitable for primary care include the Sick, Control, One stone, Fat, Food (SCOFF) tool and the Eating disorder Screen for Primary care (ESP) tool, which are short, 5-item questionnaires suitable to screen for, but not diagnose, eating disorders. Patients scoring ≥2 on either tool should be referred for further support and evaluation (Figure 2) [105,106].

Table 3 outlines key considerations before starting GLP-1-based therapy and are in alignment with Canadian Adult Obesity Clinical Practice 2020 and DNSG/EASD 2023 recommendations [51,107].

### 3.4 During the weight loss phase

The expert panel highlighted key considerations during the rapid weight loss phase of GLP-1-based therapy, with a consensus ranging from 60 to 93 % on critical recommendations and an overall alignment with recommendations by DNSG/EASD, EASO, and others (Table 4) [50,51,107,108]. Behavioural interventions, such as mindfulness and therapy-based strategies, can complement weight loss efforts [109], and should be encouraged when available. Adequate hydration, tailored to individual needs based on climate, physical activity, and health status, is critical, with recommendations ranging from 2 to 4 L/day, or approximately 35 ml water/kg bodyweight [[110], [111], [112], [113]]. Importantly, people in hot, humid environments or who perform heavy physical activity may need considerably more fluid than the recommended daily intake. The colour of the urine may be used as a practical indicator of overall hydration, with dark yellow urine indicating overall body dehydration [114]. Importantly, careful monitoring of fluid intake may be required for patients with heart failure or kidney disease [115,116].

During weight loss, decreased lean body mass naturally results from reductions in body water content, muscle mass, connective and vascular tissue, and many organ sizes. Muscle mass, specifically, falls because after weight loss, less work is required for the same level of physical activity. Unexpected or excessive weight loss (>1.5–2 kg per week) may indicate underlying chronic or inflammatory diseases, such as cancer, GI disorders, infections (all associated with significant muscle loss), or disordered eating. Such cases require careful monitoring [117].

Sufficient dietary fibre intake (≥25 g/day for women, ≥ 30 g/day for men, or ≥ 35 g/day for people with diabetes; based on a consumption of 14 g fibre per 1000 kcals in our diet) is essential for gut health, cardiometabolic benefits, and mortality risk reduction, with supplementation considered for those unable to meet these targets naturally [118,119]. Additionally, in some cases, the potent appetite reduction effect, food aversion and shift in food preferences induced by GBTs may, when accompanied by a generally low quality diet, increase the risk for nutrient deficiencies [120,121].

**Fig. 3:**
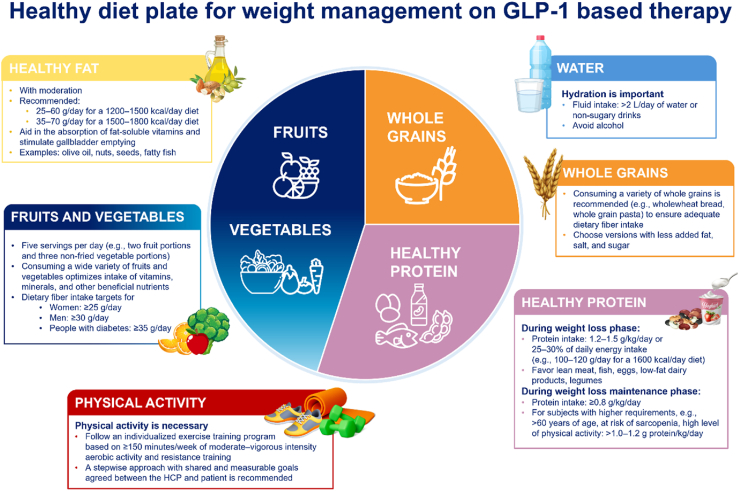
Healthy diet plate for weight management on GBT GBT, GLP-1 based therapy; HCP, healthcare professional; kcal, kilocalories; L, litre.

During the rapid weight loss phase, a protein intake of 1.2–1.5 g/kg of actual body weight/day or equivalent to 25–30 % energy on a 1600 kcal/d diet, is recommended alongside maintaining physical activity to preserve muscle mass, particularly in older adults or those at risk of sarcopenia [37,89,122,123]. Protein intake recommendations are often based on actual body weight, as they are supported by extensive research, less subjective than “ideal” or “corrected” body weight and easily understood and adjustable in clinical practice [124]. A limitation of this approach is a possible overestimation of protein needs in individuals with higher fat mass [125], therefore these recommendations may be complemented with the “plate method” (Figure 3), which is easy to apply and improves consistency [126]. Other publications recommend a protein intake of 1.0–1.5 g/kg of adjusted body weight using a specific calculation formula or 1.2–2.0 g/kg of reference or adjusted weight [127,128] or an absolute protein target of ≥ 60–75 g/day up to 1.5 g/kg body weight/day [77] or between 80 and 120 g/day [129,130]. In any case of doubt or concern, it is advised to refer the patient to a specialized, multidisciplinary centre for an in-depth evaluation which may help fine tune the GBT, nutritional and lifestyle management strategy.

Nutritionally complete low-energy formula products provide the most effective dietary approach to weight loss when used as total diet replacement (i.e., replacing all meals) for up to 12 weeks or partial diet replacement (i.e., replacing 1–2 meals/day); as meal replacements they can support weight maintenance but may require additional fibre or micronutrient supplementation [[131], [132], [133], [134]]. Recent independent systematic reviews and meta-analyses support the use of meal replacement interventions and report larger weight loss after 1 year of follow-up with meal replacements compared to conventional weight loss diets [133,135].

Importantly, individual responses to GBTs vary, and suboptimal outcomes (weight loss ≤5 % after 12 weeks of GBT) may warrant a reassessment of treatment strategies [29,136,137].

### 3.5 During the weight loss maintenance phase

Most guidelines (e.g., Obesity Canada, EASO, DNSG/EASD) recommend dietary patterns that can be used equally effectively for weight loss and maintenance, that are sustainable, affordable, and meet nutritional requirements for protein, fat, micronutrient and fibre intake [4,5,48,50,51,107,108]. Weight maintenance following initial weight loss presents challenges due to biological, behavioural, socio-economic and environmental factors driving weight regain [1,2,138]. GBT support weight stabilization after 12–18 months, with decreased meal sizes and reduced preference for energy-dense foods contributing to sustained caloric reduction [121]. Consumption of food and beverages that are rich in calories and low in nutritional value (e.g. alcohol) should be minimized. Long-term support, including counselling and tailored interventions, is essential for sustainable weight regulation [138].

Protein intake ≥ 0.8 g/kg/day is recommended, possibly more for older adults in line with the ESPEN-endorsed recommendations of at least 1.0–1.2 g protein/kg/day for healthy older people [77,91,122,123,139]. High-quality protein sources, such as lean meat, eggs, low-fat dairy products, soy, legumes and cereals should be emphasized, while the consumption of processed and saturated fat-rich meats are discouraged, as recommended across clinical practice guidelines [48,50,51,107]. Fiber intake should also remain high, incorporating both soluble and insoluble sources for cardiometabolic and GI benefits [48,50,51,107,118,119].

### 3.6 Management of common GLP-1 based therapy adverse effects

GI symptoms, such as nausea, vomiting, diarrhoea, and constipation, are the AEs most frequently reported (50–60 % of subjects) with GBT. They are dose-dependent, so usually resolve with dose reduction, and often transient because tolerance develops [22,49,140]. In addition, bowel frequency naturally falls with reduced food consumption and weight loss. A 3-year follow-up of patients treated with tirzepatide found that constipation is the most persistent GI effect during the long term [16,140]. Constipation may require proactive management through dietary fibre intake, hydration, and potentially fibre supplementation.

A mixed dietary fibre intake consisting of a combination of insoluble, soluble, and soluble viscous fibre from a variety of sources (e.g., cereals, fruit, vegetables and/or pulses) is recommended for cardio-metabolic health and to enhance digestive comfort [48,118,141]. Based on current dietary recommendations for the general population, fibre intake should be increased gradually and alongside an increased water consumption to avoid GI discomfort [142].

Both patients and healthcare professionals need to be aware of appropriate measures for avoiding and/or reducing GBT-associated GI symptoms [49,140]. Specific dietary recommendations such as small meal portion sizes, mindfulness to stop eating once full, avoiding eating when not hungry, avoiding high-fat or spicy foods (particularly during the dose titration period), minimizing intake of alcohol and fizzy drinks (especially in case of nausea or dyspepsia), and maintaining adequate hydration and fibre intake may help relieve mild-to-moderate GI symptoms [49,140,143]. Furthermore, clinical observations suggest that pharmaceutical treatment of nausea does not interfere with weight loss outcomes, as GLP-1-sensitive neuronal circuits regulating satiety and nausea appear independent [[144], [145], [146]].

### 3.7 In case of GLP-1 based therapy discontinuation

Current recommendations emphasize that GBT should not be discontinued when a stable, lowered weight is reached, unless effective alternative measures for weight loss maintenance are in place. The clinical studies STEP-4 (semaglutide), SURMOUNT-4 and the 3-year extension of SURMOUNT-1 (tirzepatide), showed that as long as GBT is maintained, weight loss is likely to be maintained, whereas patients who discontinued the GBT treatment regained around two-thirds of their weight loss within one year [16,[147], [148], [149]]. In the RCT STEP-4, a 6.9 % body weight re-gain was seen at Week 68 when patients previously treated with semaglutide were switched to placebo, compared to a further 7.9 % weight loss in patients continued on semaglutide [147]. Similar patterns of weight re-gain after stopping semaglutide were observed in the STEP-1 extension study [150].

In SURMOUNT-4, patients switched from tirzepatide to placebo at Week 36 experienced a 14.0 % weight -regain at week 88 whereas patients continued on tirzepatide experienced a further 5.5 % reduction in weight at week 88 [148].

Patients using these medications for disease modification are strongly advised to continue the therapy to support weight loss maintenance. In real life however, some individuals may need to stop treatment due to pregnancy, health issues such as traumatic accidents, before elective surgery, or during prolonged recovery from surgery; or due to financial constraints or medication access barriers [148,[150], [151], [152], [153], [154], [155], [156]]. In these cases, monitoring for weight regain is necessary, and active support should be provided for diet control. Evidence-based dietary advice, behavioural therapy, and multidisciplinary support may all help to minimize the impact of weight rebound after GBT treatment is stopped [131,147,150,157,158]. Referral to a registered dietitian is recommended as part of continued, long-term multidisciplinary team support aimed at preventing clinically significant weight regain and weight cycling [79,86].

Although the evidence is mixed, several clinical trials have demonstrated that high-protein dietary patterns may help prevent weight regain after lifestyle intervention [159], and a calorie-controlled, protein-rich diet may therefore offer benefits for preventing weight regain. Nutritionally complete low-energy formula products can be used as meal replacements to prevent weight regain, but supplementation with additional fibre and/or micronutrients may be required in some cases [131,132].

An individualized physical activity program based on ≥150 min of moderate to vigorous intensity aerobic activity and 2–3 sessions of resistance training per week, if possible, is recommended to maintain muscle function and support active weight loss [84,160]. A strategy combining exercise and GBT improves healthy weight loss maintenance more than either treatment alone [84,86]. In most cases, due to metabolic adaptation after weight loss, additional exercise (i.e., ≥ 200 min per week) may be required to prevent weight regain [161]. However, it must be acknowledged that increasing physical activity may not be an option for all people with obesity. Some people living with obesity may experience exercise as more challenging than individuals without obesity. With a BMI above 30 kg/m^2^ and excess adiposity, it can be very hard to increase physical activity. Moreover, attempts to increase physical activity without proper supervision may cause muscle and other soft-tissue injuries for people living with obesity, as many already have muscle injuries and arthritis.

## 4 Discussion

To the best of our knowledge, these are the first international Delphi consensus recommendations developed to support the GBT weight loss journey, from before starting a GBT, during the weight loss and weight management phases, and in case of GBT discontinuation. Consensus was built on a two-round modified Delphi study, resulting in the development of 52 statements outlining key considerations for the practical management of obesity with GBT, with a focus on nutrition and physical activity. This paper was then generated with several iterative rounds of editing and robust discussion among the expert group.

Importantly, our findings, which were developed by an international group of experts from countries with different healthcare systems and GBT patient access requirements, are closely aligned with the recommendations of a 2025 joint advisory from the American College of Lifestyle Medicine, the American Society for Nutrition, the Obesity Medicine Association, and the Obesity Society [129], which did not report a consensus-based approach.

A comprehensive treatment strategy with GBTs should aim to reduce adiposity, mitigate obesity-related complications, preserve muscle mass, ensure hydration and reduce common GI effects through nutritional therapy, patient education and regular physical activity [36].

To date, the effects of GBT on muscle mass are inconclusive due to small effect sizes and heterogeneity between studies (e.g., subject characteristics, study duration, body composition measurements, nutritional intake) and lack of long-term follow-up studies [36,155]. However, a recent meta-analysis of dual-energy X-ray absorptiometry-acquired body composition outcomes showed that approximately 30 % of the total weight loss achieved with GBT is attributed to lean mass loss [162], and a post hoc analysis of SURMOUNT-1 has reported that approximately 25 % of the weight loss in SURMOUNT-1 was due to lean body mass loss [163], thus suggesting that approximately 1 in 4 kg lost with GBT amounts to lean body mass loss. Numerous factors could contribute to a decline in lean mass with weight loss, and many of the cited studies did not in fact measure muscle mass or distinguish it from other components of lean mass such as organs, bone, or body water. There have been recent questions in the media and some are concerned about loss of muscle mass during weight loss under GBT [36,38,164]. People with obesity may already have relatively low muscle mass, due to factors such as aging, reduced physical activity, presence of chronic and inflammatory diseases, so loss of muscle strength would indeed be worrying. However as noted above, some loss of muscle mass is inevitable and natural with substantial weight loss, without necessarily impairing strength or activity capacity. Current evidence with GBTs does not consistently indicate excess loss of muscle mass or strength that would require additional management steps [164]. Nonetheless, several subgroups such as older age, severity of obesity, diabetes, and post-menopausal women might be particularly susceptible if there is accelerated muscle mass decline [37,89,164]. Given these diverse factors, obesity care needs to be comprehensive, sustainable and individualized to meet each patient's values, preferences and treatment goals.

Aside from clinically indicated reasons such as managing AEs or pausing/de-escalation for excessive weight loss, there is consensus by the authors that GBT should not be discontinued due to ongoing health benefits. Recently published retrospective cohort studies from the USA and Denmark using electronic health records and user surveys have reported that between 50 and 75 % of GBT users had stopped their treatment by one year [20,21,45,165]. Loss of access was the most frequent reason by far (e.g. cost, loss of insurance coverage, drug shortage); only 18 % stopped because their weight target was reached [20,21,165]. To reflect real-life clinical situations, our recommendations have encompassed the case of GBT discontinuation.

Future research should deepen our understanding of the dietary requirements of people with obesity that are undergoing treatment with GBT and future nutrient stimulated hormone therapies that produce similar changes in dietary intake and body weight. More direct evidence on nutritional interventions should be generated, such as defining protein and other macronutrient requirements, or nutritional management strategies; defining, preventing and correcting micronutrient deficiencies; and the optimization of diet and physical activity recommendations to maximize the effectiveness of GBT for weight loss. While current evidence does not indicate disproportionate loss of muscle with GBT [37,162,164], long-term follow-up studies are required. Further research is also needed on how substantial weight loss affects bone and skeletal muscle strength and function; the effect of high protein intake on bone and skeletal muscle mass and function; diet and lifestyle interventions that may allow for GBT on-and-off cycling (“stop and go” type approaches with GBT used as initial therapy prior to diet and lifestyle interventions and then as boosters) for long-term weight management and stabilization [166]. Current unmet needs also include early identification of factors associated with poor responders and strong responders to GBT; strategies for sustainable long-term GBT treatment and the prevention of weight regain after stopping GBT.

## 5 Limitations

This consensus statement has several limitations that warrant careful consideration. First, the scope of the initial literature search was restricted to a specific date range, which may have excluded more recent publications or emerging evidence. Second, the expert panel was predominantly composed of individuals from Western countries, which may have introduced regional and cultural biases into the consensus process and limited the global generalizability of the recommendations. Third, our consensus was informed by limited and poorly reported evidence regarding nutritional and physical activity interventions in the context of GBT with most randomized trials providing insufficient information to reproduce or distinguish between different lifestyle interventions. This finding is supported by a recent scoping review of 129 randomized trials of liraglutide, semaglutide, and/or tirzepatide which found minimal detailed reporting on nutritional behavior components, diet quality, or food intake [167]. As a result, the experts had to rely heavily on existing evidence and guidelines for nutrition therapy from the fields of obesity medicine or bariatric surgery and learnings from deep clinical experience. Fourth, next-generation GBTs still undergoing clinical development were excluded from this analysis, preventing consideration of potential future advancements in the field. Finally, there is a notable paucity of clinical data from non-Western regions and underrepresented ethnic populations, which could hinder the applicability of the findings across diverse patient demographics and underscores the need for more inclusive research efforts in GBT obesity management.

## 6 Conclusion

In summary, these international consensus recommendations are intended to help support all healthcare professionals involved with obesity management to achieve a healthy and sustained weight loss journey before, during and after treatment with a GBT. To date, minimal research has been reported on the impact of GBT on nutrient intake and body composition. There is also limited evidence on the effect of different dietary approaches in the context of GBT. Although no specific dietary pattern has been shown to be more effective than others, consistent encouragement and support from medical and other staff for a healthful diet composition that results in an energy deficit in the context of GBT is likely to help. There is an urgent need for more evidence to support the best medical nutrition management prior to starting therapy, during the weight loss and weight loss maintenance phases, and following discontinuation of GBT. Until more evidence becomes available, this guidance offers a multi-disciplinary expert consensus on nutrition and lifestyle interventions that support existing guidance, with much of the evidence derived from indirect evidence and clinical experience. The weight loss journey is a highly emotional one and for most patients, and GBT therapy may be a positive and empowering opportunity by helping them take control of their hunger and food cravings and by supporting them to effectively implement effective long-term strategies for nutrition and lifestyle management.  

Key takeaway clinical messages:

• GBT should be combined with a structured and individualized nutritional and lifestyle management approach that aligns with the individual's values, preferences, and treatment goals.
• Effective management of overweight/obesity with GBT involves a comprehensive strategy proactively addressing nutritional insufficiencies, increasing physical activity, managing GI symptoms, and ensuring long-term adherence to treatment. Weight management should involve support from a registered dietitian.
• Treatment monitoring and follow-up should include regular assessment of dietary intake, hydration and physical activity. Comprehensive patient support should be provided throughout the weight loss journey, encompassing pre-treatment preparation, continuous assistance during both the active weight loss and maintenance phases, as well as guidance in the event of potential therapy discontinuation.

## Author contributions

Study design and funding: JLS, LvG.

Literature review: all authors.

Data generation: all authors.

Writing and editing of the manuscript: all authors.

## Disclosures

**John Sievenpiper** reports personal honoraria from Nestlé Health Science for expert panel meetings related to project. No payment for manuscript development. Nestlé Health Science funded project management and medical writing support and reimbursed meeting travel expenses. He has received grants from public and industry sources, consulting fees from Tate & Lyle, Brightseed, and others; speaker fees from multiple food and nutrition organizations; and non-financial support from various commodity boards. **Luc Van Gaal** reports personal honoraria from Nestlé Health Science for expert panel meetings related to project. No payment for manuscript development. He has received consulting fees from Regeneron for participation in an advisory board, honoraria from Novo Nordisk for lectures, and speaker fees from Eli Lilly and Boehringer Ingelheim. **Albert Lecube** reports personal honoraria from Nestlé Health Science for expert panel meetings related to project. No payment for manuscript development. He has received speaker honoraria from AstraZeneca, Boehringer Ingelheim, Eli Lilly, Novo Nordisk, and Pronokal; travel support from Sanofi, Novo Nordisk, Menarini, and Lilly; and served on advisory boards for Boehringer Ingelheim, Eli Lilly, Nestlé Health Science, Novo Nordisk, and Pronokal. He has received research funding from Amgen, AstraZeneca, Boehringer Ingelheim, Eli Lilly, Novo Nordisk, Pfizer, and other public institutions.

**Andreas Pfeiffer** reports personal honoraria from Nestlé Health Science for expert panel meetings related to project. No payment for manuscript development. He has received grants from multiple EU and national research bodies; consulting fees from Abbott, Berlin Chemie, Novo Nordisk, Wörwag, and Nestlé; speaker fees from various pharmaceutical companies; and advisory roles with the Deutsche Diabetes Stiftung. **Angela Fitch** reports personal honoraria from Nestlé Health Science for expert panel meetings related to project. No payment for manuscript development. She has received speaking honoraria from Eli Lilly, Novo Nordisk, Currax, and Rhythm; served on advisory boards for Neurobo, Abbvie, Eli Lilly, Novo Nordisk, and Currax; owns stock in Eli Lilly and Novo Nordisk; and received a body composition scale from Seca for an advisory role. **Bettina Mittendorfer** reports personal honoraria from Nestlé Health Science for expert panel meetings related to project. No payment for manuscript development. **Donna Ryan** reports personal honoraria from Nestlé Health Science for expert panel meetings related to project. No payment for manuscript development. She has received consulting fees from multiple companies including Altimmune, Amgen, AstraZeneca, Eli Lilly, Novo Nordisk, and others; honoraria from Lilly and Novo Nordisk; travel support; and advisory roles with Lilly, Rhythm, and CinRX. She holds stock options in several healthcare-related companies. **Jamy Ard** reports personal honoraria from Nestlé Health Science for expert panel meetings related to project. No payment for manuscript development. He has received grants from health and nutrition organizations; consulting fees from companies including Nestlé, Eli Lilly, Novo Nordisk, and others; and received materials or support from KVK Tech, WW, and Nestlé Healthcare Nutrition. **John B. Dixon** reports personal honoraria from Nestlé Health Science for expert panel meetings related to project. No payment for manuscript development. He has received consulting fees from Rechape Lifescience and Nestlé; lecture honoraria from Novo Nordisk, Lilly, and others; travel support; and has served on advisory boards for Reshape Lifesciences, Nestlé Health Science Australia, Lilly, and Novo Nordisk. **Kamlesh Khunti** reports personal honoraria from Nestlé Health Science for expert panel meetings related to project. No payment for manuscript development. He has received consulting and speaker fees from numerous pharmaceutical companies including Amgen, AstraZeneca, Lilly, Novo Nordisk, Sanofi, Pfizer, and Nestlé. **Linda Gigliotti** reports personal honoraria from Nestlé Health Science for expert panel meetings related to project. No payment for manuscript development. She has also received travel support, consulting fees from Amgen, and honoraria from dietetic and nutrition associations. **Matthias Blüher** reports personal honoraria from Nestlé Health Science for expert panel meetings related to project. No payment for manuscript development. He has received consulting and speaker fees from Amgen, AstraZeneca, Bayer, Boehringer Ingelheim, Lilly, Novo Nordisk, Novartis, and Sanofi, and served on a Boehringer Ingelheim advisory board. **Mike Lean** reports personal honoraria from Nestlé Health Science for expert panel meetings related to project. No payment for manuscript development. He has received fees for lectures and training from Novo Nordisk, Eli Lilly, and Nestlé; departmental research grants from NIHR and MRC; and consulting fees from Counterweight paid to his institution. **Tina Vilsbøll** reports personal honoraria from Nestlé Health Science for expert panel meetings related to project. No payment for manuscript development. She has served on scientific advisory panels and speaker bureaus for Amgen, AstraZeneca, Boehringer Ingelheim, Eli Lilly, Novo Nordisk, and other companies. **Wei Chen** reports personal honoraria from Nestlé Health Science for expert panel meetings related to project. No payment for manuscript development. He declares no other financial relationships.

## Declaration of Artificial Intelligence (AI) and AI-assisted technologies

During the preparation of this work the authors did not use AI.

## Source of funding

This work was supported by Nestlé Health Science.
