# Supplementary material for: Nutritional and lifestyle supportive care recommendations for management of obesity with GLP-1 - based therapies: An expert consensus statement using a modified Delphi approach
Source: Obes Pillars. 2025 Nov 11;17:100228. doi: 10.1016/j.obpill.2025.100228 (PMC12768930; doi:10.1016/j.obpill.2025.100228)
Supplement: Multimedia component 1 [file mmc1.docx]

**Supplement**

## Supplement table 1. Search terms used for the scoping literature search using the date range January 1, 2021 to June 30, 2025 in PubMed, Embase, Web of Science, Cochrane, and Medline.

| **Step​** | **Query ​** | **Search terms​** |
| --- | --- | --- |
| 1​ | Search 1​ | “Glucagon-like peptide-1” OR “GLP-1 agonists” OR “GLP-1 receptor agonists” OR “Incretin mimetics” OR “GLP-1 analogs” OR “Semaglutide” OR “Liraglutide” OR “Dulaglutide” OR “Tirzepatide” AND “Weight loss” OR “weigh control” OR “weight management” OR “weight reduction” OR “loss of weight” OR “decrease in body mass” OR “weight loss maintenance” OR “weight rebound” OR “weight gain” OR “weight re-gain” ​ |
|  | Search 2​ | “Glucagon-like peptide-1” OR “GLP-1 agonists” OR “GLP-1 receptor agonists” OR “Incretin mimetics” OR “GLP-1 analogs” OR “Semaglutide” OR “Liraglutide” OR “Dulaglutide” OR “Tirzepatide” AND “Obesity” OR "Overweight" OR "BMI >=25" OR "BMI ≥25"​ |
| 2​ | X.1​ | [Search 1 or Search 2 terms] AND “Gastro-intestinal side effects” OR “Gastrointestinal side effects” OR “GI side effects” OR “gastrointestinal adverse events” “diarrhea” OR “diarrhoea” OR “nausea” OR “constipation” OR “fatigue”​ |
|  | X.2​ | [Search 1 or Search 2 terms] AND “Body composition” OR “Lean body mass” OR “LBM” OR “fat free mass” OR “muscle mass”​ |
|  | X.3​ | [Search 1 or Search 2 terms] AND “sarcopenia” OR “elderly” OR “sarcopenic” OR “frailty” OR “frail”​ |
|  | X.4​ | [Search 1 or Search 2 terms] AND “Active lifestyle” OR “exercise” OR “sport” OR “physical activity” ​ |
|  | X.5​ | [Search 1 or Search 2 terms] AND “Malnutrition” OR “Nutrition” OR “nutritional” OR “nutrient intake” OR “intake of nutrients” OR “macronutrients” OR “micronutrients” OR “food supplement” OR “vitamin” OR “protein intake” OR “protein” OR “dietary fat” OR “fiber/fibre” OR “minerals” OR “carbohydrates”​ |
|  | X.6​ | [Search 1 or Search 2 terms] AND “Dietary patterns” or “therapeutic diet” or “low calorie diet” ​ |
|  | X.7​ | [Search 1 or Search 2 terms] AND “Digital” OR “telehealth” OR “patient support” OR “app” OR “patient programme” OR “patient program"​ |
|  | X.8​ | [Search 1 or Search 2 terms] AND “treatment termination” OR “treatment discontinuation” OR “dose reduction”​ |

## Supplement table 2. Physical exercise regimens used in GBT RCTs [83, 84]

| **Group exercise** | **Individual exercise** |
| --- | --- |
| - Twice weekly:   - 30 minutes of vigorous-intensity, interval-based indoor cycling aimed at achieving ≥80% maximal heart rate, and   - 15 minutes of circuit training combining vigorous-intensity aerobic exercise and muscle-strengthening exercises using bodyweight or external resistance (e.g., 3 circuits of 5 exercises over 40 seconds each followed by 20 second breaks) | - Twice weekly moderate-to vigorous– intensity exercise, such as:   - Cycling   - Running   - Brisk walking   - Individual circuit training |

GBT, GLP-1 based therapy; RCT, randomised controlled trial

## Supplement table 3. Similarities and differences between GLP-1 weight loss expert Delphi recommendations and the 2025 Joint Advisory (ACLM/ASN/OMA/TOS) guidance on GBT for weight loss.

|  | **GLP-1 weight loss expert Delphi recommendations** | **Joint Advisory (ACLM/ASN/OMA/TOS) guidance [129]** |
| --- | --- | --- |
| Methodology | Delphi methodology with multiple voting rounds | Methods not reported. Recommendations based on literature review |
| Expert composition | Experts from the European Union, Canada, Australia, USA and China | Experts from USA and Canada |
| Focus on access and affordability | No | Yes |
| Focus on behavioural interventions | No | Yes |
| Focus on nutrition in obesity | Yes | Yes |
| Focus on physical activity and reduction of lean body mass loss | Yes | Yes |
| General nutritional recommendations for people with obesity | Yes | Yes |
| General physical activity recommendations for people with obesity | Yes | Yes |
| Examples of RCT-supported specific exercise regimens in conjunction with GBT weight loss | Yes | No |
| Strategy for screening for disordered eating prior to starting GBT | Yes | No |
| Recommendations for before starting GBT for weight loss | Yes | Yes |
| Recommendations for GBT weight loss phase | Yes | Yes |
| Recommendations for GBT weight maintenance phase | Yes | Yes |
| Considerations to reduce lean body mass loss during GBT | Yes | Yes |
| Recommendations for GBT side effect management | Yes | Yes |
| Recommendations for managing stopping GBT for weight loss | Yes | No |

ACLM, American College of Lifestyle Medicine; ASN, American Society for Nutrition; GBT, GLP-1 based therapy; GLP-1, glucagon-like peptide 1; OMA, Obesity Medicine Association; RCT, randomised controlled trial; TOS, The Obesity Society; USA, United States of America.
